# Accuracy and completeness of patient pathways – the benefits of national data linkage in Australia

**DOI:** 10.1186/s12913-015-0981-2

**Published:** 2015-08-08

**Authors:** James H. Boyd, Sean M. Randall, Anna M. Ferrante, Jacqueline K. Bauer, Kevin McInneny, Adrian P. Brown, Katrina Spilsbury, Margo Gillies, James B. Semmens

**Affiliations:** Centre for Population Health Research, Faculty of Health Sciences, Curtin University, Bentley, 6102 WA Australia

## Abstract

**Background:**

The technical challenges associated with national data linkage, and the extent of cross-border population movements, are explored as part of a pioneering research project. The project involved linking state-based hospital admission records and death registrations across Australia for a national study of hospital related deaths.

**Methods:**

The project linked over 44 million morbidity and mortality records from four Australian states between 1st July 1999 and 31st December 2009 using probabilistic methods. The accuracy of the linkage was measured through a comparison with jurisdictional keys sourced from individual states. The extent of cross-border population movement between these states was also assessed.

**Results:**

Data matching identified almost twelve million individuals across the four Australian states. The percentage of individuals from one state with records found in another ranged from 3-5 %. Using jurisdictional keys to measure linkage quality, results indicate a high matching efficiency (F measure 97 to 99 %), with linkage processing taking only a matter of days.

**Conclusions:**

The results demonstrate the feasibility and accuracy of undertaking cross jurisdictional linkage for national research. The benefits are substantial, particularly in relation to capturing the full complement of records in patient pathways as a result of cross-border population movements.

The project identified a sizeable ‘mobile’ population with hospital records in more than one state. Research studies that focus on a single jurisdiction will under-enumerate the extent of hospital usage by individuals in the population. It is important that researchers understand and are aware of the impact of this missing hospital activity on their studies.

The project highlights the need for an efficient and accurate data linkage system to support national research across Australia.

## Background

### Administrative data as a research tool

Administrative datasets are a powerful resource enabling health researchers to answer epidemiological questions that require long-term follow up on large samples of the population [1]. Access to administrative collections such as hospital records, health registries and birth and death information enables research which would otherwise be very expensive and organisationally difficult to undertake [2].

To allow researchers to gain a picture of an individual’s health over time, data linkage techniques are utilised to identify which administrative records from multiple datasets belong to the same person. This process allows the researcher to answer questions about the health of individuals over time, rather than solely about discrete health events [3].

Data linkage has several advantages over other study methods. It is far less intrusive and costly than collecting the same information by other means, such as through large-scale surveys. It allows entire populations to be studied, reducing common problems with follow-up encountered in survey based research designs [4]. Its shortcomings lie in the inflexibility of the data (only information already recorded can be used for analysis). Data linkage studies can also face issues regarding loss to follow up; individuals can move out of a catchment area under study, for instance. The extent of this loss to follow up, and its effect on research results, is largely unknown.

### Data linkage methods and linkage quality

In the absence of a unique identifier, data linkage is carried out using demographic information such as name, date of birth and address. As these identifiers can change and be in error (or contain missing information), probabilistic statistical methods are used to ensure the highest quality of linked data [5].

Two types of errors impact linkage quality: false positives, where two records are designated as a match when they should not be, and false negatives, where two records are designated as a non-match when they should not be. The rate of these two errors, measured through precision (or positive predictive value) and recall (sensitivity) statistics, determines overall linkage quality [6].

Ensuring high linkage quality is difficult and typically requires manual efforts. Organisations involved in routine, large-scale data linkage frequently employ a system of manual review of created links to monitor and maintain linkage quality [7, 8]. This can be time and resource intensive, and some errors can still exist even after review. As datasets become larger, the cost and time of manual review becomes prohibitive.

### Linkage infrastructure in Australia

Data linkage facilities exist in many parts of the world including Australia, the UK and Canada [4, 9–12]. Australia has been a pioneer in the development of linkage infrastructure for research. Western Australia (WA) has operated a linkage unit since 1995, while the Centre for Health Record Linkage (CHeReL) in New South Wales (NSW) has been in operation since 2006 [13].

From 2009, there has been significant additional government investment in expanding the data linkage research infrastructure in Australia [14]. The creation of a “cross-jurisdictional” linkage capability (that is, the ability to link data from more than one state or territory) was a key component of the Population Health Research Network (PHRN) initiative established under the National Collaborative Research Infrastructure Strategy [15, 16]. Given the federated nature of healthcare service delivery in Australia (that is, some services are delivered and administered at state level, while others are delivered and administered at Commonwealth level), cross-jurisdictional linkage is an essential component of national infrastructure. Without cross-jurisdictional data linkage capabilities, research aimed at national level or targeting issues of common interest (e.g. health service use along border areas) cannot be undertaken. Research at a national level also has other benefits, such as increased statistical power, and reduced loss to follow up caused by interstate movement.

Several ‘Proof of Concept’ (POC) collaboration projects were initiated by the PHRN to demonstrate the feasibility of moving large datasets across the country, linking these to a high quality in a short period of time, and using the subsequent linked data to answer research questions of national importance [16].

The first of these POC collaborations linked hospital admissions records with death data across several states, focusing on deaths occurring in hospital or within 30 days of hospitalisation. The project was the first of its kind in Australia.

### Study aims

The purpose of this paper is twofold. Firstly, to highlight the technical achievements associated with undertaking data linkage for this first POC collaboration.

The paper intends to show that national linkage of ‘big data’ can be carried out efficiently and accurately. As well as scalable linkage services, an effective national linkage infrastructure needs to deliver high quality linkage results. Current methods for ensuring high linkage quality rely heavily on manual processes, which are not feasible on large datasets. For national linkage to be viable, high linkage quality must be achieved and maintained through automated methods alone.

The second aim of the paper is to demonstrate the importance and impact of cross-jurisdictional linkage. The study will capture population movement at individual or person-based level through linkage of disparate datasets, enabling researchers to assess the full extent of health service utilisation across state borders. The effect of more complete patient pathways on research outcomes has not been previously documented and is not well understood. With reliable estimates of cross-border population flows and service utilisation, researchers can gain a better picture of the need for national linkage studies over state-based linkages projects.

## Methods

### Datasets and ethics approvals

**Fig. 1:**
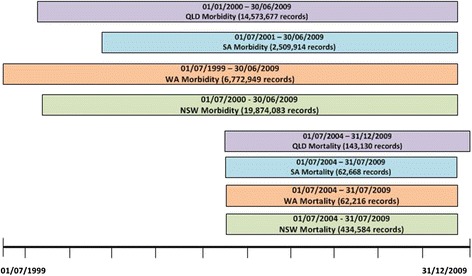
Datasets provided for proof of concept collaboration

The data for the POC collaboration included up to ten years of state-based hospital admissions and mortality records from four Australian states between 1st July 1999 and 31st December 2009: Western Australia (WA), New South Wales (NSW), South Australia (SA) and Queensland (QLD) (see Fig. 1). Hospital data was supplied from both public and private hospitals in WA, NSW and QLD; at the time of the project, only admissions from public hospitals in SA were available for linkage. Ethical approval for this study was obtained from Human Research Ethics Committees in WA Health, QLD Health, SA Health, the Cancer Institute NSW and Curtin University (WA).

A total of 44,433,221 records were provided for linkage. In keeping with the separation principle [17], only demographic information was supplied for linkage [16]. Each record comprised information on the person’s full name, sex, date of birth and address, as well as admission and separation dates for hospital events (or date of death, for mortality events). Over 30 % of NSW and QLD hospital records did not contain any name information, these records were sourced from private hospitals which did not permit the disclosure of this information. Table 1 provides a summary by state and data collection of the missing data within the variables supplied for linkage.

**Table 1:** Percentage of missing data in linkage variables

Linkage Variables	NSW		WA		SA		QLD	
	Hospital	Mortality	Hospital	Mortality	Hospital	Mortality	Hospital	Mortality
Family name	31.9 %	<0.1 %	<0.1 %	<0.1 %	5.3 %	<0.1 %	34.7 %	<0.1 %
Given name(s)	33.9 %	<1.0 %	<1.0 %	<1.0 %	5.5 %	<0.1 %	36.4 %	<0.1 %
Sex	<0.1 %	<0.1 %	<0.1 %	<0.1 %	<0.1 %	<0.1 %	<0.1 %	<0.1 %
Date of Birth	<0.1 %	<0.1 %	<0.1 %	<1.0 %	<0.1 %	<0.1 %	<0.1 %	<0.1 %
Address	7.5 %	<0.1 %	<1.0 %	2.9 %	8.1 %	<1.0 %	<0.1 %	<0.1 %
Suburb	<1.0 %	1.7 %	<0.1 %	<1.0 %	6.9 %	<1.0 %	<0.1 %	<1.0 %
Postcode	<1.0 %	1.3 %	<1.0 %	<1.0 %	8.5 %	<1.0 %	<0.1 %	4.0 %

As WA and NSW had well established linkage infrastructure in place, records from these states had been linked and extensively reviewed *within* their own jurisdiction and assigned a jurisdiction-specific linkage key. These linkage keys identified which records within a particular state belonged to a person within that state. Using these jurisdictional keys, it was possible to directly compare our linkage quality results with those from each of these jurisdictions.

### Linkage strategy

Probabilistic linkage methods were used for matching, owing to their flexibility and simplicity [18, 19]. Notwithstanding the size of the datasets, this matching process involved a series of comparisons between two records and a decision as to whether they belong to the same individual. The matching process included a ‘blocking’ step which limited comparisons to those records which share a minimum level of identifying information. This was important with the large datasets as the potential number of comparisons would be too large to process without the blocking step.

A set of blocking variables were defined for the project [18] and only records which agreed on one of these blocks were compared. The linkage strategy involved two blocks, the first used phonetic surname code (soundex) in combination with first initial and the second selected record pairs for comparison on date of birth and sex [6].

The matching step involved comparing all demographic variables in each blocked pair of records. Each comparison had an associated weight based on the specific agreement and disagreement information provided by individual variables. These variable weights were based on the probability that two values agreed on a record pair given that the two records belong to the same person and the probability of two records belonging to different people when they had the same value.

Agreement and disagreement weights were estimated using knowledge from previous linkages, and refined further in a number of pilot linkages. After computing these weights, a pair comparison score was created by summing agreement and disagreement weights across the demographic variables. If the comparison score for a pair of records exceeded a specified threshold, it was deemed a match [18].

All available demographic variables were used for comparison. Alphabetic variables were compared using the Jaro-Winkler string comparator [20] which computes a score based on the similarity of the strings. Year of birth was scored on a graded scale, receiving a higher score the closer the values were to each other. All other comparisons were based solely on whether the values exactly matched or not.

All datasets were linked to all other datasets, and each dataset was also internally linked. Linkages were initially performed without reference to the provided jurisdictional linkage keys so as to measure linkage quality against these.

### Linkage quality

Of primary interest in measuring linkage accuracy is the number of true matches and non-matches identified as links and non-links. To evaluate linkage quality, three standard metrics were used: precision, recall and F-measure [21].

Precision refers to the proportion of returned links that are true matches. It is sometimes referred to as positive predictive value. Recall is the proportion of all true matches that have been correctly linked. Recall is also known as sensitivity. The F-measure of a linkage is the harmonic mean between precision and recall. This provides a single figure with which linkage quality can be compared.

These metrics have been highlighted as suitable for measuring data linkage quality [22, 23] and have been used in evaluations of linkage software [6].

Following the assessment of linkage accuracy, a series of automated and semi-automated procedures were used on the patient based record groups to identify and resolve errors. These included algorithms which addressed groups with multiple deaths, hospital records after death as well as unusually large groups (i.e. groups with more than 5000 records).

### Linkage efficiency

As a cross jurisdictional project, which involved data files with large number of records, it was not feasible to compare all possible record pairs to establish links. Instead a series of blocks were employed which aimed to reduce the number of comparisons without having an impact on linkage quality (i.e. reduce comparisons without missing ‘True Positive’ links). To assess the efficiency and quality of the blocks we calculated two complexity metrics, the reduction ratio and pairs completeness score [24].

The reduction ratio provided an assessment of the decrease in comparisons as a result of the blocking strategy. This was calculated as the ratio of actual blocked comparisons to the total possible comparisons and measured the efficiency of the strategy without measuring the impact on linkage quality.

The percentage of ‘true pairs’ blocked or pairs completeness metric measured the number of true positive pairs compared in the blocking strategy as a percentage of all possible true positive pairs identified using the jurisdictional linkage keys for WA and NSW records. Records from these states were used as they have been linked and extensively reviewed *within* their own jurisdiction.

There is an obvious balance between the reduction ratio and percentage of ‘true pairs’ blocked. If the comparisons are reduced for efficiency it can have an impact on linkage quality and increasing comparisons to maximise quality can significantly impact the time required to process the linkage. The blocking strategy is therefore the reference point for all additional linkage quality estimates (i.e. precision and recall).

## Results

Over 44 million records across morbidity and mortality collections were linked within and between each jurisdiction. The linkage strategy produced a series of records pairs each with a matching score which were used to identify records belonging to an individual across all data sources. The linkage strategy was evaluated in terms of blocking efficiency and linkage quality.

### Blocking efficiency

Using the blocking strategy outlined, approximately 142 billion comparisons were performed during the linkage process. These matching assessments made up only 0.014 % of all possible record pairs from the full comparison space. The blocking process was similar within each jurisdiction, with the state-based reduction ratio ranging between 0.99973 and 0.99987. Table 2 provides a summary of the matching comparisons undertaken.

**Table 2:** Blocking efficiency

Linkage Comparison Summary		NSW	WA	SA	QLD	Total
Number of records supplied for linkage:						
	Hospital	19,874,083	6,772,949	2,509,914	14,573,677	43,730,623
	Mortality	434,584	62,216	62,668	143,130	702,598
	Total	20,308,667	6,835,165	2,572,582	14,716,807	44,433,221
Linkage comparison space:						
	Blocked Comparisons	26,071,726,251	6,328,711,086	821,279,963	13,597,405,294	142,112,536,420
	Reduction Ratio	0.99987	0.99973	0.99975	0.99987	0.99986
	Possible Pairs Blocked (%)	0.0126 %	0.0271 %	0.0248 %	0.0126 %	0.0144 %
	‘True’ Pairs Blocked (%)^a^	99.76 %	99.95 %	-	-	-

^a^‘True’ pairs based on the jurisdictional linkage key supplied by WA and NSW

### Linkage accuracy

Linkage results were compared against those produced by state-based linkage units in WA and NSW (both these datasets were supplied with a jurisdictional linkage key). The jurisdictional links from these states were used as a gold standard and allowed an evaluation of linkage quality against each individual state (that is, comparing within-state results only).

The accuracy results for all linkages were exceptionally high with over 99.76 % of all ‘true pairs’ made available for comparison through blocking i.e. a very small number of pairs identified by WA and NSW jurisdictional linkage keys were lost as a result of the blocking strategy (Table 2). This provided a baseline for assessing the linkage quality of all blocked comparisons.

In WA, over 99.9 % of the morbidity pairs identified as links were found to be correct, and 98.1 % of all possible within-jurisdiction morbidity links were found. This resulted in a maximum F-measure quality score of 0.99 where 1.000 would indicate a perfect linkage (see Table 3) indicating ‘an average’ error rate for morbidity data from these jurisdictions of less than 1 %.

**Table 3:** Linkage quality

Jurisdictional Data	NSW			WA
	Morbidity	Public	Private	Morbidity
Accuracy of national linkage:				
Precision	0.988	0.994	0.983	0.999
Recall	0.963	0.996	0.917	0.981
F-measure^a^	0.976	0.995	0.949	0.990

^a^F-measure is the harmonic mean of precision and recall

One factor which had an effect on both blocking and matching accuracy was missing data in the linkage variables (Table 1). Over 30 % of NSW hospital records did not contain any name information (these records were sourced from private hospitals which did not permit release of this information). As a consequence, the quality results for our linkages on WA data were higher than that of NSW. The linkage of morbidity records in NSW provided an overall F-measure of 0.976 (precision = 98.8 % and recall = 96.3 %).

NSW results were further disaggregated by hospital status (public versus private). Records from public hospitals showed much higher results (F-Measure = 0.995) indicating that the lack of demographic information accounted for the drop in linkage quality (Table 3).

### Patient summary statistics

The final results of the linkage across the various jurisdictions are summarised in Table 4. Across the four jurisdictions almost 12 million individuals accounted for the 44 million records. Under half (45 %) of the individuals identified with hospital records had a single hospital admissions record; with the remainder having an average of 5.9 hospital records per person.

**Table 4:** Patient summary results

Linkage Results - Summary		NSW	WA	SA	QLD	Total
Number of individuals:						
	Identified from Hospital and Death records	5,796,784	1,558,999	848,446	3,995,812	11,954,874
Hospital events within individual groups:						
	Number of individuals hospitalised	5,782,670	1,554,313	833,781	3,979,562	11,907,114
	Singleton hospital records^a^	2,598,149	544,484	433,277	1,831,768	5,407,678
	%	44.9 %	35.0 %	52.0 %	46.0 %	45.4 %
	Maximum number of hospital records	2,297	2,245	2,393	2,393	2,393
	Average group size^b^	5.4	6.2	5.2	5.9	5.9

^a^Individuals who only have one hospital record in their group

^b^Singletons are not included in the total number of individuals for this calculation

The number of individuals with a single hospital record varied across the four jurisdictions with Western Australia (WA) having the smallest proportion (35 %) and South Australia (SA) having the highest (52 %). Similarly, the average group size (i.e. the record per individual) varied between 6.2 and 5.2 in WA and SA respectively. It should be noted that the South Australian figures do not include private hospital records which may influence the proportion of singleton groups in that state.

Cross-border population movements and hospital usage statistics over the study period are summarised in Table 5. The proportions of individuals in each state with records in one or more of the other three states were classified as a ‘mobile’ population. The ‘mobile’ population was largest in QLD with 5 % of individuals having hospital records in other states and lowest in SA and WA where 3 % were classified as ‘mobile’ individuals. The ‘mobile’ population accounted for between 4 and 7 % of the episodes of care in each state jurisdiction.

**Table 5:** Patient mobility

		NSW	WA	SA	QLD
Population mobility or cross-border flows (over study period)					
	Mobile population^a^	205,551	47,575	29,645	202,859
	% of individuals in that state	4 %	3 %	3 %	5 %
	Static population^b^	5,591,233	1,511,424	818,801	3,792,953
	% of individuals in that state	96 %	97 %	97 %	95 %
Number of events					
	Mobile population	1,135,905	248,480	137,234	1,014,912
	% of jurisdiction records	6 %	4 %	5 %	7 %
	Static population	19,172,762	6,586,685	2,435,348	13,701,895
	% of jurisdiction records	94 %	96 %	95 %	93 %

^a^Mobile population refers to the number of individuals in a jurisdiction/state that have records in other states

^b^Static population refers to the number of individuals in a jurisdiction/state that have records *only* in that state

## Discussion

The linkage described here was part of a large POC collaboration that tested the efficiency and accuracy of newly established national data linkage infrastructure in Australia.

### Linkage quality

The accuracy and efficiency of the linkage was shown to be high with a large number of ‘blocked’ pairs comparisons removed from the matching process with very little impact on the linkage quality. Using validated linkage information from WA and NSW, little discrepancy was found between the created links and those found by jurisdictional linkage units in those states. The existence of some discrepancies can be attributed to the additional quality work carried out by those jurisdictional linkage units. Jurisdictional linkage units in Australia typically employ extensive manual review of created links, along with stringent regular manual quality checks. Further errors are identified through feedback following the use of the linked data in research projects. Some of the difference in results could also be attributed to the limited number of identifiers supplied for cross-jurisdictional linkage. Linkage quality depends heavily upon the quality of the underlying dataset. NSW data, with one third of names missing, had the lowest overall linkage quality using our linkage strategy (without additional data collections or clerical intervention).

These quality comparisons rely on the use of jurisdictional linkages as the gold standard. These links from WA and NSW have been validated by researchers who have used them widely. In addition, significant expertise has been developed by these organisations which have a long history of linkage. Having access to two entire sets of extensively checked links allowed us to gain a very accurate estimate of our quality. Few previous investigations into linkage quality have had such a reliable and large gold standard with which to test their results. Typical measures of linkage quality have used samples of links to gain an estimate of quality, often able only to estimate the number of incorrect links created, with the number of links missed essentially unknown [25], or have used relative measures to estimate missed links [26] which allows relative comparison, but not absolute quality measures.

### Cross border population movement

Linking hospital records across four states over a ten year time span showed that, on average, between 3 % and 5 % of patients within one state had hospital record in another state. The results further showed that between 4 % and 7 % of hospital records occurring in a state can be attributed to an individual who also has records in another state.

These findings suggest that research studies examining patient pathways may underestimate the total number of event records belonging to individuals if they do not factor in cross-border hospital admissions. In studies involving hospital admissions events from a single state, it is important that researchers are aware of the incomplete nature of information and the impact this may have on research outcomes. The size and impact of this underestimation will depend on several factors such as the selection of study cohort and the study period, with longer study periods being more susceptible to population movement into and out of the jurisdiction.

It has been shown that data linkage quality can have an overall impact on research outcomes, potentially biasing results [27]. However, incomplete patient pathways as a result of cross-border flows are not often addressed in linked epidemiological research. When a significant proportion of patients are having hospital activity in more than one jurisdiction, it is important that researchers understand the impact of this incomplete information on single jurisdiction studies [28]. The impact of this data omission on research outcomes is uncertain and warrants further research into the effect of linkage quality and incomplete patient pathways on research outcomes.

## Conclusion

These results show the feasibility of large scale data linkage infrastructure, producing high quality results through efficient linkage processes. Overall, data linkage quality in large scale linkage remains very high, despite the lack of stringent manual quality review procedures, which would be extremely costly on datasets of this size. Importantly, this type of linkage identifies cross-border population movement, enabling researchers to fully describe patient pathways.

The national linkage infrastructure has been successfully used to join together records from multiple administrative datasets which belong to the same person. The infrastructure has been developed to be flexible and scalable, addressing the traditional challenges and limitations of efficiently linking national data. With an increasingly ‘mobile’ population with life event records in different states, this “cross-jurisdictional” linkage service will have positive benefits on Australian health research.
